# Hereditary Behavior for Center Segregation and Inclusions in Q355 Steel Slabs with Ti and Nb Addition

**DOI:** 10.3390/ma18174157

**Published:** 2025-09-04

**Authors:** Keke Tong, Ya Gao, Houxin Wang, Zhong Huang, Guoxi Wan, Dajiang Zhang, Xiurong Zuo

**Affiliations:** 1 Key Laboratory of Materials Physics, Ministry of Education, School of Physics, Zhengzhou University, Zhengzhou 450001, China; 2 CITIC Metal Co., Ltd., Beijing 100004, China; gaoya@citic.com (Y.G.); wanghx@citic.com (H.W.); 3 Henan Iron and Steel Group Co., Ltd., Zhengzhou 450046, China; hz1222@126.com (Z.H.); ailinyu032@163.com (D.Z.); 4 Anyang Iron and Steel Co., Ltd., Anyang 455004, China; wanguoxi-8888@163.com

**Keywords:** Q355 steel, slab, center segregation, inclusion, Mn, Ti, Nb

## Abstract

This paper investigates the effects of Ti and Nb addition with varying Mn content on the solidification macrostructure and microstructure in the continuous casting slab of Q355 steel using optical microscopy, scanning electron microscopy, transmission electron microscopy, and electron probe microanalysis. The evolution of central segregation and MnS inclusions during thermal simulation compress deformation has been clearly established using Gleeble-1500 thermal simulation tester. The results indicate that by reducing the Mn content and adding a small amount of Ti and Nb, it is possible to refine the grain and mitigate the center segregation of Q355 steel. Mn steel with 1.25% Mn and without Ti and Nb addition exhibits the most severe center segregation. The TiNb steel with 0.52% Mn and a small amount of Ti and Nb addition showed a marked improvement in the center segregation of the slab. The Nb steel with 0.56% Mn and 0.009% Nb shows the presence of thin film ferrite along prior grain boundaries surrounded by Widmanstätten ferrite, and the central segregation has not shown significant improvement. The thermal simulation samples of the three steel types inherit the characteristics of their respective casting structures.

## 1. Introduction

Low-alloy structural steels have been widely used in construction, bridges, and machinery manufacturing due to its excellent properties and relatively low cost [1,2]. Center segregation is a primary macroscopic defect in continuous casting billets affecting the uniformity of microstructure and mechanical properties of the material [3,4,5,6]. During the continuous casting process, the redistribution of solute elements at the solid–liquid interface and the movement of the interface toward the center of the slab lead to continuous enrichment of solute elements at the slab center, ultimately resulting in center segregation [7,8]. Due to the difficulty in eliminating central segregation during subsequent hot rolling or heat treatment processes, the degradation of the properties of steel plates was inevitable. However, by adjusting composition, increasing cooling rates, lowering casting temperatures, and employing final electromagnetic stirring techniques, central segregation can be alleviated or eliminated [6,9,10,11,12]. For low-alloy structural steels, to mitigate center segregation, adjusting the alloy composition may be a more direct and effective approach with low cost.

Mn is a key alloying element which can enhance the strength by means of solid solution strengthening in low-alloy steels. Furthermore, Mn would react with S to form stable MnS inclusions in steel, avoiding the formation of FeS. Due to the low melting temperature of FeS, cracking would occur during thermo-mechanical treatment. However, present research indicates that Mn can result in center segregation [13,14]. In addition, with Mn increase, the hazards of MnS inclusion increase, which disrupts the uniformity of the microstructure. However, the reduction in Mn content leads to the decrease in solid solution strengthening effect. Consequently, careful regulation of Mn content is crucial for optimizing the microstructure and properties of steel.

The addition of microalloying elements can significantly enhance the mechanical properties of steel through the solid solution, fine grain strengthening, and precipitation strengthening, which can compensate for the loss in strength due to the reduced Mn content [15]. At present, there is extensive research on the influence of Ti and Nb microalloying elements on the microstructure and properties of steel. It is found that Ti and Nb are capable of forming nanoscale precipitates, which exhibit excellent grain refinement and precipitation strengthening effects [16,17,18], improving hardness, toughness, wear resistance, and thermal stability [19,20,21]; the common addition of Ti and Nb demonstrates a synergistic effect for grain refinement and precipitation strengthening [22,23]. Pan et al. [24] found that a small amount of Ti addition could form TiN at high temperature, while normal content of Al formed AlN at low temperature, failing to prevent the grain coarsening during heating, unless Al content was increased as high as 0.07%. Moreover, with the Ti and Nb addition, non-recrystallization critical temperature was increased, expanding the temperature range of non-recrystallization rolling during hot rolling, which facilitated the microstructure refinement [25].

Present studies indicate that both Ti and Nb improve not only the mechanical performance of steel but also the microstructure uniformity. However, the influence of Ti and Nb addition on center segregation and hereditary behavior for the center segregation between slab and plate have not been clearly established.

In this study, the effects of Ti and Nb on the microstructure and mechanical properties of Q355 steel were primarily investigated. It involves a comparative analysis of the macrostructure, microstructure, inclusions, and hardness of cast samples with varying contents of Mn, Ti, and Nb, as well as the evolution of central segregation and MnS inclusions during thermal simulation compress deformation. The findings aim to provide guidance for mitigating center segregation in continuous casting slabs and enhancing the overall quality of steel.

## 2. Materials and Methods

**Figure 1:**
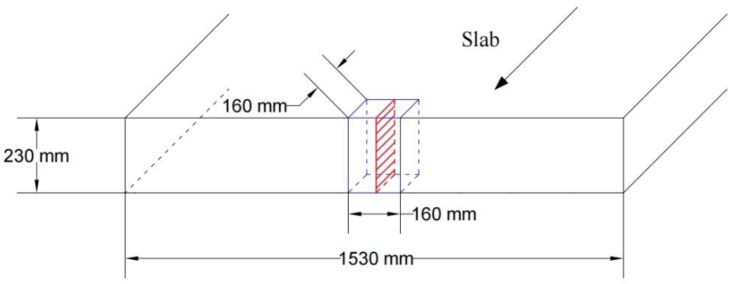
Sampling schemes in continuous casting slab.

In this study, Q355 steel was cast by a vertical bending continuous caster; its composition is shown in Table 1. The test steels were categorized into three types: steel sample with 1.25% Mn was labeled as Mn steel, steel sample with 0.56% Mn, 0.009% Nb and trace amounts of 0.002% Ti introduced by the raw materials during steelmaking was labeled as Nb steel, and steel sample with 0.52% Mn, 0.009% Nb, and 0.015% Ti was labeled as TiNb steel. The slabs had a width of approximately 1500 mm and a thickness of about 230 mm. Slab samples with sizes of 160 mm × 160 mm × 230 mm were taken from center in width, as shown in Figure 1. The red shaded area represented the longitudinal observation surface for the inclusion observation. The samples were mechanically polished and then etched with a 4% nitric acid alcohol solution. The microstructure and inclusions in slab thickness direction were observed and analyzed using optical microscopy (OM; Axiolab 5, ZEISS, Oberkochen, Germany), scanning electron microscopy (SEM; JSM 6700F, JEOL, Tokyo, Japan), Energy-dispersive spectroscopy (EDS; INCA-ENERGY, Oxford, UK), and Electron Probe Micro-Analysis (EPMA; EPMA-8050G, Shimadzu, Kyoto, Japan). Microhardness tests were conducted along the full thickness direction of the cast slab, at intervals of 0.5 mm between the two points. The test force applied was 1 kgf, and the holding time was 15 s (XHD-2000TMSC, Vickers, Shanghai, China). The 3 mm thin foil sample was prepared using a double-jet electro-polishing apparatus (TenuPol-5, Struers, Ballerup, Denmark) and subsequently observed under a transmission electron microscope (TEM; Tecnai F20, FEI, San Jose, CA, America).

**Figure 2:**
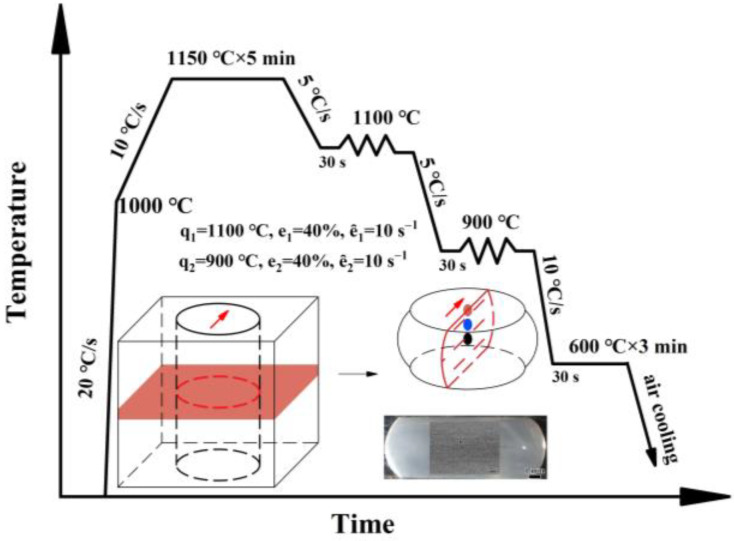
Thermal simulation experiment scheme.

Thermal simulation samples with sizes of Φ 8 mm × 14 mm were cut from the slab thickness center including the central segregation. The thermal simulation plan is shown in Figure 2, which included double-pass compression using Gleeble-1500 thermal simulation tester (Gleeble1500D, DSI, Lockport, IL, USA). The first pass compression was carried out at 1100 °C, while the second pass compression was carried out at 900 °C, with a compression rate of 10 s^−1^. After the thermal simulation test, the samples were cut along the longitudinal direction for OM and SEM observation, and the size and quantity of MnS inclusions in the sample center were counted by OM. Microhardness testing was conducted at the center of the longitudinal cross-section, with a test load of 0.1 kgf and a holding time of 15 s.

## 3. Results

### 3.1. Hardness and Macrostructure of Casting Slab

**Figure 3:**
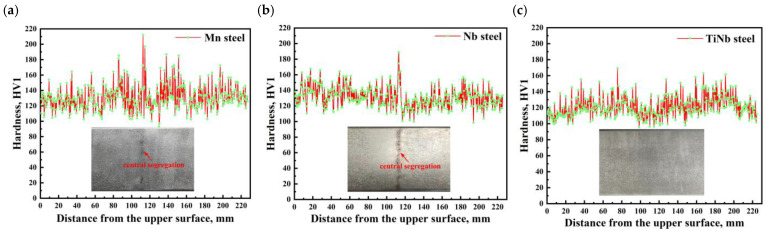
Hardness along slab thickness direction and macrostructure of continuous casting slab. (**a**) Mn steel, (**b**) Nb steel, and (**c**) TiNb steel.

Hardness and macroscopic morphology in the thickness direction of the continuous casting slab are displayed in Figure 3. Significant hardness fluctuation in the slab thickness direction, and black segregation region in the slab center are found in Figure 3. However, the average hardness value for TiNb steel slab sample (approximately 120 HV1) was slightly lower than that of Mn steel and Nb steel (approximately 135 HV1) in the slab thickness direction. For Mn steel and Nb steel samples, notably, the hardness peak values at the central thickness region were observed, corresponding to the segregation regions in the slab center (inset in Figure 3). However, owing to the absence of significant central segregation in TiNb steel slab, there is no discernible hardness peak at the central thickness region, indicating superior microstructure uniformity. In conclusion, it can be observed that although the Mn content in Nb steel and TiNb steel (0.56% Mn and 0.52% Mn, respectively) was obviously reduced, compared with Mn steel (1.25% Mn), the hardness values did not obviously decrease. This indicated that the addition of Nb and Ti elements had a strengthening effect. Furthermore, the addition of Nb and Ti had improved the central segregation of slab, leading to a more uniform hardness distribution [26].

### 3.2. Microstructure of Casting Slab

**Figure 4:**
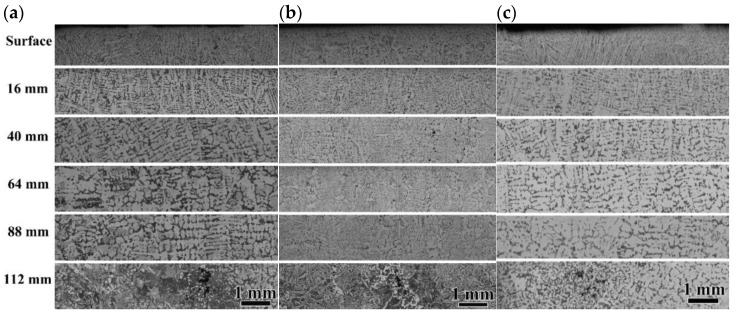
OM images of slab in thickness direction of Mn steel (**a**), Nb steel (**b**), and TiNb steel (**c**).

Figure 4 presented the microstructure of longitudinal surface of the casting slab. Notable segregation regions were observed at the central thickness regions of Mn steel and Nb steel slab samples, accompanied by irregularly cavities within segregation regions. The presence of such cavities in casting slab reduced the effective load-bearing area, and led to stress concentration, which may serve as potential sources for crack initiation [5]. In contrast, TiNb steel sample exhibited a mild degree of central segregation.

**Figure 5:**
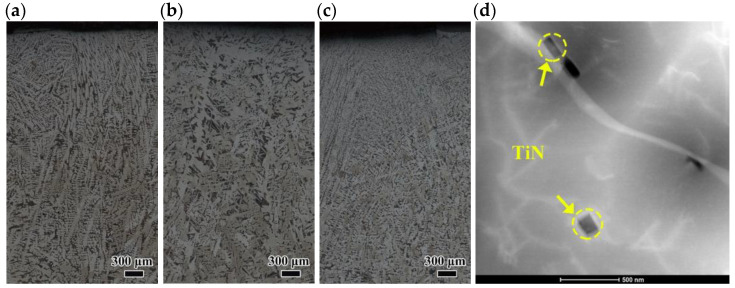
OM and TEM images of the surface of the slabs. (**a**) Mn steel, (**b**) Nb steel, and (**c**,**d**) TiNb steel.

Figure 5 illustrates the microstructural morphology of slab surfaces in the thickness direction. From Figure 4 and Figure 5, it was found that the microstructure from the surface to the center of the casting slabs consisted of pearlite and ferrite. The Mn steel specimen exhibited well-developed dendrites and a notable uneven aggregation of pearlite. Additionally, there were pronounced segregated zones at the center, characterized by the presence of voids surrounded by coarse pearlite structures along with a small amount of ferrite. For Nb steel, besides the severe central segregation zones and voids, a significant presence of inhomogeneous microstructure including film-like ferrite along the prior grain boundaries were observed. The sole addition of Nb did not obviously refine the grain structure. In contrast, the TiNb steel specimen demonstrated the most uniform microstructure in the thickness direction of the slab, with slight pearlite segregation at the center. Simultaneous addition of Ti and Nb would lead to a pronounced precipitate strengthening effect and fine grain strengthening effect, further enhancing the overall mechanical properties of the steel [22].

As shown in Figure 5, the Mn steel specimen exhibited well-developed dendrites on the surface, without significant film-like ferrite present along the prior grain boundaries. On the contrary, the Nb steel specimen featured the pronounced film-like proeutectoid ferrite along the grain boundaries (GB ferrite), surrounded by a significant amount of coarse Widmanstätten ferrite with the three-dimensional shape of elongated spikes or triangular cones [27]. Widmanstätten ferrite nucleated on the GB ferrite surface, which was separated by a large-angle GB between them, whereas a small-angle GB presented at the interface of the Widmanstätten sideplates, as well as the interface of the sideplate and other grains [28]. Furthermore, according to Wang et al. [29], the strong Nb and C segregations at GB occurred during the casting process, which resulted in long strips of Nb carbonitride forming in the GB ferrite during cooling process [30]. During plastic deformation, stress concentrations can easily develop at the interface of long strip-shaped Nb carbonitride and GB ferrite, as well as the interface of Widmanstätten sideplate and pearlite, leading to surface crack formation [31]. In contrast, the TiNb steel featured fine and uniform equiaxed grains on the surface with about 2 mm thickness. The pin effect of the nanometer-sized TiN in the grain boundary kept the grain boundary from sliding, which resulted in grain refining (Figure 5d).

In summary, the Mn steel exhibited well-developed dendritic structures and severe central segregation. The sole addition of Nb in Nb steel had not shown the marked improvement in the central segregation. In addition, a film-like ferrite structure was observed at grain boundaries surrounded by Widmanstätten ferrite, which introduced a risk of crack formation. In contrast, the simultaneous addition of Nb and Ti in TiNb steel refined the grains and achieved a more uniform distribution of pearlite, leading to substantial improvements in surface grains and central segregation.

### 3.3. Central Segregation and Cavity of Casting Slab

**Figure 6:**
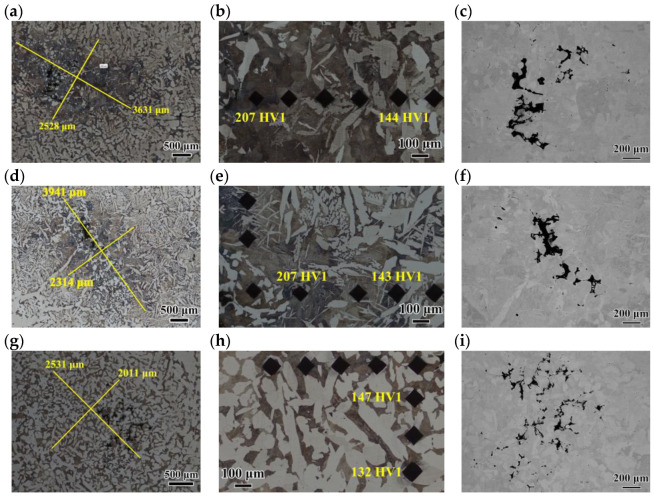
OM images and SEM images of central segregation of Mn steel (**a**–**c**), Nb steel (**d**–**f**), and TiNb steel (**g**–**i**).

An aggregation of cavity at the central segregation region of the casting slabs was formed by solidification shrinkage and was not supplemented by the solute-enriched liquid phase at the end of solidification (Figure 6c,f,i). Mn steel exhibited the largest cavity size among three types of steel. These cavities were usually welded during the subsequent rolling process. However, if the cavity sizes were excessively large, they may fail to be welded, which served as a source of cracking. Figure 6a,d,g reveal that the central segregation featured the increased fraction of the pearlite surrounding the cavities, owing to solute enrichment. In Nb steel, the extent of pearlite segregation was reduced. At the same time, there existed allotriomorphic ferrite along the prior austenite grain boundaries and acicular ferrite within the pearlite. In comparison, TiNb steel showed a relatively dispersed cavities, whose size was significantly smaller than those in Mn steel and Nb steel. And, the pearlite and ferrite being uniformly distributed, exhibited a slight difference between the segregation region and the adjacent areas. In a word, owing to the decrease in segregating element Mn and the addition of Ti and Nb with obvious grain refining effect, TiNb steel featured the dispersed cavities and refining pearlite colony. In conclusion, there existed significant difference in the central segregation and cavity among the three types of steel, which probably arose from different amounts of Mn, Ti, and Nb in steel.

Microhardness testing was performed in the segregation regions and adjacent non-segregated regions of the three slab samples (testing load of 1 kgf). In Mn steel and Nb steel, the variations in hardness indentation size were quite significant (Figure 6b,e). The pearlite was predominant in the central segregation area, whose hardness was higher than that of ferrite. So, the hardness gradually decreased away from the segregation region. In contrast, TiNb steel exhibited the slight microstructure difference between the segregation area and adjacent area, so the differences in hardness were relatively small (Figure 6h).

### 3.4. Inclusions in Casting Slab

**Figure 7:**
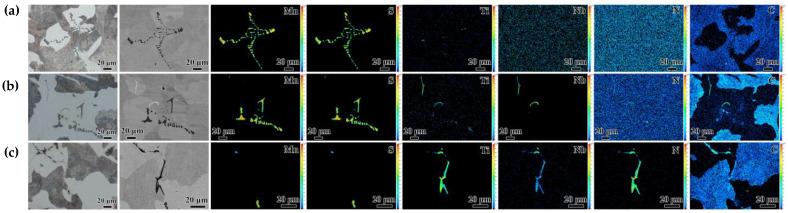
OM images and EPMA mapping images in the thickness center of the slab. (**a**) Mn steel, (**b**) Nb steel, and (**c**) TiNb steel.

The type, size, and amount of the inclusions have a significant impact on the mechanical properties of steel. Therefore, the precipitation behavior of TiN/NbC and MnS was studied in this paper. In the thickness center of slab in Mn steel, the typical morphologies of MnS inclusions -were dendritic, and a small amount of rodlike or blocky (Figure 7a). As shown in Figure 7b, two types of inclusions were observed in the central segregation region in the Nb steel, which were a large amount of MnS and a small amount of NbC/TiN with a low content of Ti. As shown in Figure 7c, a large amount of irregularly shaped orange-red TiN/NbC with a relatively high content of Ti were observed, whereas the typical morphologies of MnS inclusions were mainly rodlike or blocky. When the central segregation zones occurred grain refining, the size of MnS and TiN inclusion were also refined, due to less space for MnS to grow [32]. It was also found that almost all inclusions were on the ferrite, and large-sized MnS and TiN inclusion usually assembled in the central segregation zone.

**Figure 8:**
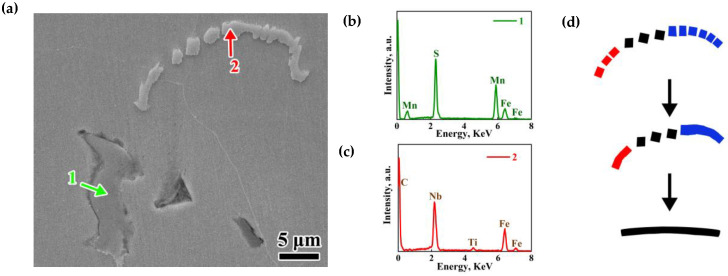
Inclusions in casting slab. (**a**) SEM, (**b**,**c**) EDS of inclusions, and (**d**) schematic drawing of aggregation of NbC-TiN.

Figure 8 presents the SEM image of various morphologies of inclusions observed at the center of the slab in Nb steel. Both blocky MnS (green arrow) and chain-like NbC-TiN (red arrow) inclusions were identified by EDS. Nb steel sample exhibited a small amount of NbC-TiN with low Ti content. In TiNb steel sample, a significant increase in TiN-NbC inclusions with significant amounts of Ti was noted at the central segregation zone. NbC-TiN appeared in a chain-like arrangement (Figure 8a). This phenomenon was due to the aggregation of small-sized TiN particles at higher temperature, and successively Nb (C, N) precipitated on TiN at lower temperature. The TiN was not fully connected, and finally an uncontinuous chain-like structure was formed.

### 3.5. Thermodynamic Calculations

**Figure 9:**
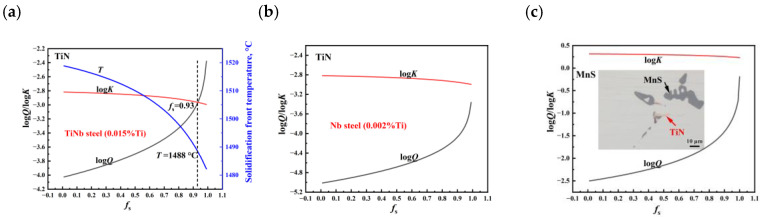
(**a**) Relationship between the *K*_TiN_/*Q*_TiN_/*T* and *f*_s_ in TiNb steel. (**b**) Relationship between the *K*_TiN_/*Q*_TiN_ and *f*_s_ in Nb steel. (**c**) Relationship between *K*_MnS_/*Q*_MnS_ and *f*_s_, and OM image of inclusions.

Figure 9c illustrates the different morphologies of TiN and MnS inclusions in the casting slab. It can be observed that MnS either covered the TiN or grew at the edges of TiN, indicating a close symbiotic relationship between them. Thermodynamic calculations were performed to analyze the precipitation behavior of the TiN and MnS inclusions.

The chemical reaction equation for the formation of TiN and MnS is represented as follows:

(1) $$Ti+N={TiN}_{inclusion}$$

(2) $$Mn+S={MnS}_{inclusion}$$

The theoretical solubility product of TiN (*K*_TiN_) and MnS (*K*_MnS_) under the liquid phase can be expressed as follows:

(3) $$\log K_{TiN}=\log\left[Ti\right]\cdot \left[N\right]=-\frac{15218}{T}+5.64$$

(4) $$\log K_{MnS}=\log\left[Mn\right]\cdot \left[S\right]=-\frac{6890}{T}+4.16$$

The temperature of the solidification front (*T*) can be expressed as

(5) $$T=T_{Fe}-\frac{\left(T_{Fe}-T_{l}\right)}{1-f_{s}\cdot \frac{\left(T_{l}-T_{s}\right)}{\left(T_{Fe}-T_{s}\right)}}$$

where the melting point temperature of molten steel (*T*_Fe_) was 1537 °C; the liquidus temperature (*T*_l_) was 1519 °C; and the solidus temperature (*T_s_*) was 1481 °C. JMatPro (Version 7.0.0) software was used to obtain *T*_l_ and *T_s_*. *f*_s_ is the solid fraction.

The actual concentration product of TiN (*Q*_TiN_) and MnS (*Q*_MnS_) in the molten steel is as follows:

(6) $$Q_{TiN}=\frac{{{\left[Ti\right]}_{0}\cdot {\left[N\right]}_{0}\cdot \left(1-f_{s}\right)}^{k_{Ti}-1}}{1-\left(1-k_{N}\right)\cdot f_{s}}$$

(7) $$Q_{MnS}=\frac{{{\left[Mn\right]}_{0}{\cdot [S]}_{0}\cdot \left(1-f_{s}\right)}^{k_{Mn}-1}}{1-\left(1-k_{s}\right)\cdot f_{s}}$$

where [Ti]_0_, [N]_0_, [Mn]_0_, and [S]_0_ represented their initial concentrations of Ti, N, Mn, and S, respectively. *k*_Ti_ and *k*_N_ represented the equilibrium distribution coefficients of Ti and N, which was 0.33 and 0.48, respectively. The equilibrium distribution coefficients of Mn (*k*_Mn_) and S (*k*_S_) were 0.785 and 0.035, respectively.

According to Figure 9a, for TiNb steel with 0.015% Ti, TiN began to precipitate when *f*_s_ was 0.93 in the solid–liquid two-phase region, with a precipitation temperature of 1488 °C. However, according to Figure 9b, in Nb steel with trace 0.002% Ti, TiN precipitated below the solidus temperature. Despite the very low residual Ti content, a small amount of Ti-containing precipitates can still be occasionally detected.

MnS precipitated below the solidus temperature, as shown in Figure 9c. According to the theory of lattice mismatch, the one-dimensional mismatch formula as described by Equation (8) can be used to calculate the mismatch (*δ*) between materials with the same lattice structure [33]. The lattice parameter of TiN is 0.424 nm, and that of MnS and Al_2_O_3_ is 0.53651 nm and 0.476 nm, respectively. The mismatch of TiN-MnS and Al_2_O_3_-MnS is 0.2097 and 0.1128, respectively, indicating that TiN and Al_2_O_3_ were more likely to be an heterogeneous nucleation core for MnS, which was consistent with results established by the first principle method [34]. With continuous growth, MnS inclusions connected or wrapped TiN inclusions subsequently (Figure 9c). Therefore, MnS and TiN inclusion assembled in the central segregation zone due to the solute enrichment. With the reduction in Mn content from 1.25% in Mn steel to 0.52% in TiNb steels, as well as the S content from 0.011% in Mn steel to 0.006% in TiNb steels, accompanied by obvious grain refining due to Ti and Nb addition, the quantity and size of MnS diminished significantly, thereby mitigating the detrimental effects of central segregation [32,35]. In a previous study by Ma et al. [23], TiN served as a substrate for the epitaxial growth of NbC, promoting the precipitation of NbC.

(8) $$\delta =\frac{\left|a_{m}-a_{n}\right|}{a_{n}}$$

where *a*_m_ and *a*_n_ represented the low index surfaces lattice constant of substrate and nucleation core, respectively.

### 3.6. Thermal Simulation Test

**Figure 10:**
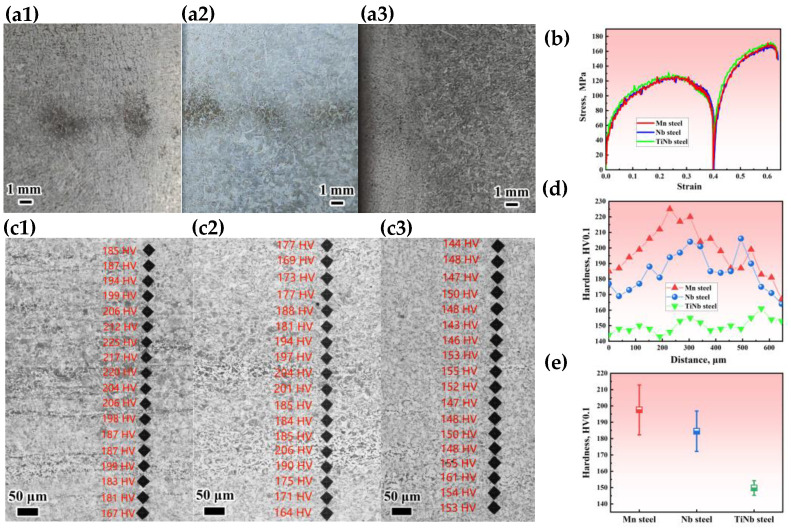
Macroscopic morphology of the specimens before thermal compression of Mn steel (**a1**), Nb steel (**a2**), TiNb steel (**a3**), and true stress–strain curve (**b**). OM images of microhardness indentation (HV0.1) in thermal simulation sample center of Mn steel (**c1**), Nb steel (**c2**), TiNb steel (**c3**), hardness distribution (**d**), and hardness mean value and standard deviation (**e**).

Samples were extracted from the central segregation regions of the casting slabs in Mn steel, Nb steel, and TiNb steel for double-pass compression thermal simulation tests to replicate the hot rolling process, aiming to investigate the hereditary behavior of central segregation and inclusions during hot rolling process. Figure 10(a1–a3) showed the macroscopic longitudinal cross-section of samples before the thermal simulation tests. The Mn steel and Nb steel specimens exhibited the serious center segregation after thermal simulation tests (Figure 10(c1,c2)), while the TiNb steel specimen showed imperceptible segregation regions (Figure 10(c3)), which inherited the central segregation of each slab, respectively. Figure 10b presented the true stress–true strain curves during the thermal simulation. The curves for the three specimens were nearly overlapping, indicating that the steels with the addition of Nb and Ti had similar recrystallization behavior in the first stage and work hardening behavior in the second stage of the thermal compression process, without an increase in the deformation resistance required for rolling.

Figure 10(c1–c3) presented the microhardness indentation maps of the central segregation region of the thermal simulation samples (testing load of 0.1 kgf). The Mn steel and Nb steel specimens exhibited distinct segregation regions, where the hardness was higher than those in the adjacent non-segregated areas (Figure 10(c1,c2),d,e). In contrast, the TiNb steel sample exhibited a uniform microstructure in its central region, resulting in slight fluctuations in hardness values (Figure 10(c3),d,e).

**Figure 11:**
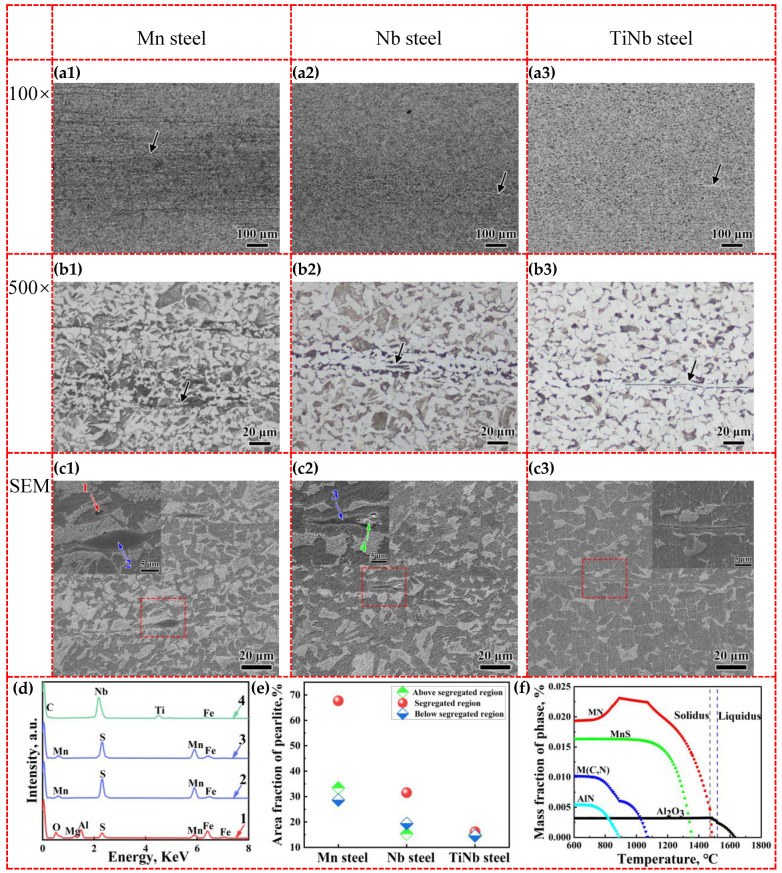
OM images and SEM images of Mn steel (**a1**,**b1**,**c1**), Nb steel (**a2**,**b2**,**c2**), and TiNb steel (**a3**,**b3**,**c3**), EDS of inclusions (**d**), proportion of pearlite (**e**), and relationship between precipitate content and temperature of TiNb steel calculated using the JMatPro software (**f**).

Figure 11 presented the microstructure at the center of the thermal simulation samples. Figure 11(a1–a3) showed low-magnification OM images at the thickness center of Mn steel, Nb steel, and TiNb steel samples, respectively. The central segregation regions were approximately 400 μm wide in the Mn steel and Nb steel, while the TiNb steel sample did not display obvious central segregation. Combined with Figure 6, although the segregation region could not be eliminated in the thermal simulation compressed process, the pores within the segregation region could be welded. The pearlite fraction in the segregated area of Mn steel was significantly higher than that in the adjacent non-segregated region (Figure 11e), indicating that high Mn content made the pearlite accumulate and formed severe central segregation with high hardness compared with surrounding matrix (Figure 11(a1)). Whereas Nb steel followed with a moderate level of pearlite accumulation. In contrast, the pearlite distribution in TiNb steel was relatively uniform without distinct segregation bands, thereby resulting in a more homogeneous microstructure and hardness distribution (Figure 11(a3)). The above analysis results of thermal simulation compressed samples were consistent with slabs, showing a hereditary behavior of central segregation during the rolling process.

High-magnification OM images and SEM images (Figure 11(b1–b3,c1–c3)) revealed that the segregation regions included ferrite bands surrounded by fine pearlite bands forming sandwich structure (black arrows in Figure 11). It was observed that MnS inclusions were generally located in the white ferrite bands zone (Figure 11(c1–c3),d). In contrast to Mn steel, the band qualities in Nb steel and TiNb steel obviously decreased and pearlite on both sides of the ferrite bands were significantly refined. The microstructure of hot-rolled steel plates exhibited a hereditary relationship with those of the continuously cast slabs. During the rolling process, unevenly distributed solidification dendrites of slabs resulted in enrichment and depletion zones of solute elements [36].

The formation of MnS resulted in the low Mn concentration regions, known as “Mn depleted zone”. Mn can stabilize austenite and lower the temperature at which austenite transforms into ferrite. Therefore, in Mn-depleted zone, the transformation temperature from austenite to ferrite increases, causing ferrite to preferentially nucleate around the MnS inclusions, leading to an increase in Mn concentration in adjacent areas [10]. Cementite nucleates in Mn-rich regions, resulting in the formation of pearlite bands around ferrite band.

MnS and TiN inclusions were usually distributed within the ferrite, and TiN inclusions were wrapped by MnS (green arrow in Figure 11(c2)). MnS inclusion which wrapped the Al_2_O_3_ inclusion could also be found (red arrow in Figure 11(c1)), due to the low misfit of 0.1128, as shown in Section 3.5. As shown in Figure 11f, Al_2_O_3_ precipitated first from steel melt as deoxygenation products at 1631 °C. Secondly, TiN precipitated from solid–liquid two-phase region at 1483 °C. Then, MnS started to precipitate in austenite during the cooling process at 1356 °C. When temperature continued to decrease, NbN started to precipitate at 1071 °C. Finally, AlN precipitates at 890 °C. Combined with lattice mismatch, calculated using Equation (8), Al_2_O_3_ and TiN can be used as the heterogeneous nucleation core for MnS. Ultimately, Al_2_O_3_ and TiN were wrapped by MnS with excellent deformability, thereby facilitating the lowering of the detrimental effect of two kinds of inclusion during rolling [37].

**Figure 12:**
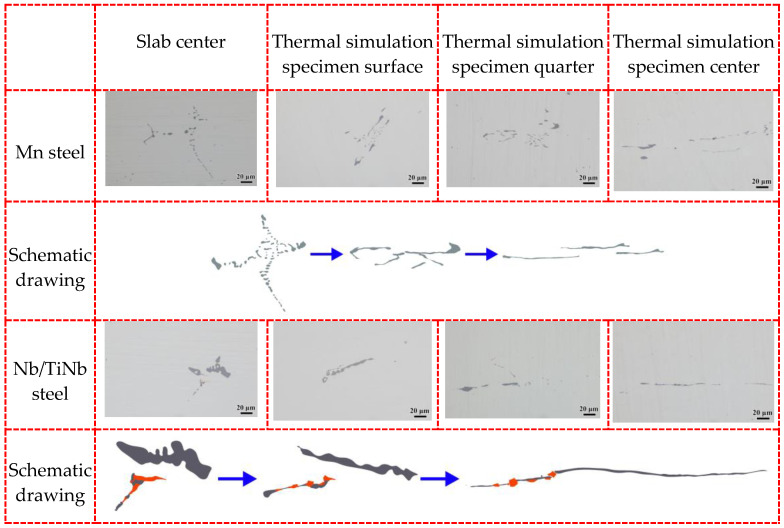
Morphology of inclusions at surface, quarter, and center in thickness direction.

The morphology of inclusions was observed at the surface, quarter, and center in the thickness direction of the thermal simulation samples corresponding to different deforming degree from small to large. As shown in Figure 12, the distribution of MnS inclusions at the surfaces of the thermal simulation sample (red mark in Figure 2) with the smallest deformation degree was similar to that of the slab. However, at the quarter position (blue mark in Figure 2), deformation along the compressing direction was observed. At the central position of the samples (black mark in Figure 2) with the largest deformation, elongated band-like MnS inclusions along the compressing direction were identified. The deformation degree of MnS inclusions gradually increased from the surface to the center of the thermal simulation samples, which reflected the changes in inclusion morphology during the rolling process. Accompanied by deformation of MnS inclusions from spherical or dendritic shapes to long strips, the voids in the central segregation were gradually welded together [36].

**Figure 13:**
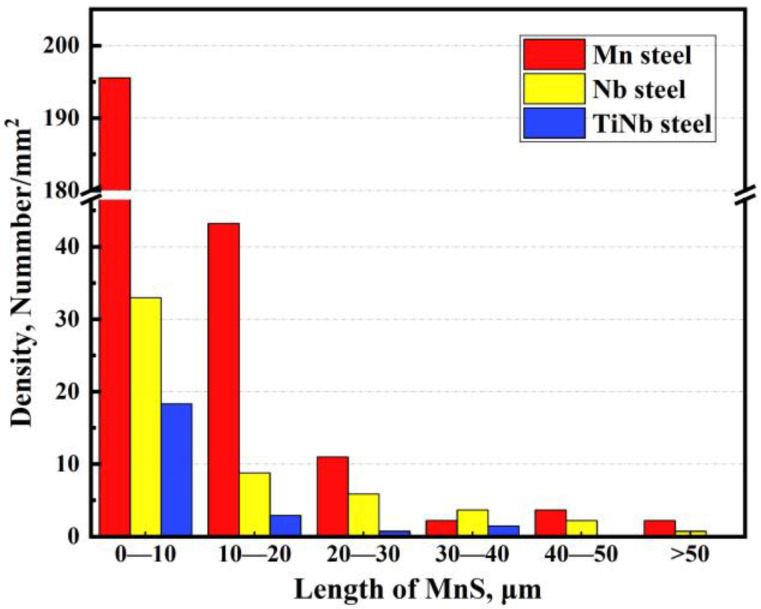
Length distribution of MnS inclusions in thermally simulated samples of Mn steel, Nb steel, and TiNb steel.

Figure 13 presents the size distribution of MnS inclusions at the center of hot simulation specimens for three types of steel. It was found that the density of MnS inclusion concentrated in the length range of <30 μm was 249.8, 47.6, and 22.0 number/mm^2^ in the Mn steel, Nb steel, and TiNb steel, respectively, while that with the length exceeding 30 μm was 8.06, 6.59, and 1.46 number/mm^2^, respectively. In a word, the significantly higher quantities of larger size MnS inclusion in the Mn steel were observed, compared to the Nb steel and TiNb steel specimens. Whereas the TiNb steel exhibited the least quantity and smallest size of MnS inclusion among the three types of steel. This trend aligns with the distribution pattern observed in the slab, demonstrating the inheritance behavior of MnS inclusions. By reducing the quantities and effectively suppressing the formation of large-sized MnS inclusions, the TiNb steel significantly mitigated the detrimental effects of MnS inclusions.

## 4. Conclusions

(1) The Mn steel exhibited well-developed dendritic structures, the most severe center segregation and cavities. The Nb steel showed the presence of thin film ferrite along prior grain boundaries surrounded by Widmanstätten ferrite, and the central segregation had not shown marked improvement with sole Nb addition. In contrast, the common addition of Ti and Nb in TiNb steel refined the grains and achieved a more uniform distribution of pearlite and cavities, leading to substantial improvements in central segregation.
(2) In the thickness center of slab in Mn steel, the typical morphologies of MnS inclusions were dendritic. In TiNb steel, a large amount of irregularly shaped TiN/NbC were observed, whereas the typical morphologies of MnS inclusions were mainly rodlike or blocky. In Nb steel and TiNb steel, the deformable MnS inclusions usually wrapped TiN/NbC inclusions, reducing the detrimental effects of TiN/NbC inclusions. Thermodynamic calculations and the results from lattice mismatch theory indicated that TiN was likely to serve as a core for MnS formation.
(3) The microstructure of double-pass compression thermal simulation steel plates exhibited a hereditary relationship with those of the continuously cast slabs. The same law was found in cast samples and thermal simulation samples, that is, the most serious center segregation occurred in Mn steel, followed by Nb steel, whereas TiNb steel without distinct segregation bands exhibited the most homogeneous microstructure and hardness distribution. Center segregation in casting slabs cannot be eliminated after double-pass compression, while the voids in the central segregation were gradually welded together as deformation increased. MnS inclusions were deformed from spherical or dendritic shapes to long strips during compression process.

## Figures and Tables

**Table 1:** The chemical composition of Q355 casting billet (wt%).

Sample	C	Si	Mn	P	S	Al	Nb	Ti	N
Mn steel	0.16	0.17	1.25	0.020	0.011	0.004	0.003	0.001	0.0054
Nb steel	0.18	0.19	0.56	0.024	0.006	0.005	0.009	0.002	0.0048
TiNb steel	0.16	0.15	0.52	0.021	0.006	0.007	0.009	0.015	0.0062

## Data Availability

The original contributions presented in this study are included in the article. Further inquiries can be directed to the corresponding author.
